# Localized Scleroderma Is Associated with an Increased Risk of Systemic Sclerosis: A Matched Cohort Study

**DOI:** 10.2340/actadv.v106.adv-2026-0759

**Published:** 2026-10-06

**Authors:** Bastian Aschoff, Gabriela Riemekasten, Sören Dräger, Ralf J. Ludwig, Philip Curman

**Affiliations:** 1 Lübeck Institute of Experimental Dermatology, University of Lübeck and University Clinic Schleswig Holstein (UKSH), Lübeck, Germany; 2 Department of Rheumatology and Clinical Immunology, University of Lübeck, Lübeck, Germany; 3 Department of Dermatology, University Clinic Schleswig Holstein (UKSH), Lübeck, Germany; 4 Institute and Comprehensive Center for Inflammation Medicine, University of Lübeck, Lübeck, Germany; 5 Dermato-Venereology Clinic, Karolinska University Hospital, Stockholm, Sweden; 6 Department of Medical Epidemiology and Biostatistics, Karolinska Institutet, Stockholm, Sweden; 7 Dermatology and Venereology Division, Department of Medicine (Solna), Karolinska Institutet, Stockholm, Sweden

Localized scleroderma (LoS) is a chronic inflammatory connective tissue disorder characterized by skin fibrosis with variable clinical presentations (1). Although LoS can cause significant cutaneous morbidity, it is generally confined to the skin and subcutaneous tissue and is not believed to progress to systemic sclerosis (SSc) (2–4). In contrast, SSc is a systemic autoimmune disease characterized by vasculopathy and fibrosis that can affect virtually any organ and carries the highest mortality among rheumatic diseases (5).

Because LoS and SSc are both fibrosing skin disorders, some clinicians have speculated that LoS may represent a cutaneous-limited form of SSc. This view is supported by the relatively frequent presence of autoantibodies or Raynaud phenomenon, typical features of SSc, in LoS (6). A recent systematic review identified coexistence of LoS and SSc in up to 11% of SSc patients, finding that SSc preceded LoS in 47% of cases, LoS preceded SSc in 26% of cases with a mean latency of approximately 6.6 years (range 6 months to 41 years), and concurrent onset occurred in the remaining 26% (7). However, only 2 studies have systematically examined whether LoS predisposes to SSc. In a retrospective single-centre cohort of 330 SSc patients, 8 had coexisting LoS, most preceding SSc onset, leading the authors to conclude that the diseases are distinct (3). Similarly, a prospective multicentre study of 76 LoS patients and 101 matched controls found no excess of SSc-related features and no progression to SSc during 8 years of follow-up (2). Together, these studies argue against frequent progression of LoS to SSc, but their limited sample sizes may have been underpowered to detect an uncommon event given the low base rate of SSc in the general population. Given this limited evidence, the true incidence and timing of SSc after LoS remains uncertain.

## MATERIALS AND METHODS

We performed a large-scale, propensity-score matched, retrospective cohort study using electronic health records (EHR) from the TriNetX real-world database (8), which aggregates data from over 150 million patients across healthcare organizations worldwide (Fig. S1). Patients with a diagnosis of LoS were identified and matched 1:1 to non-LoS controls on age, sex, race, ethnicity and a range of baseline comorbidities. Patients with a pre-existing diagnosis of SSc or Raynaud syndrome prior to the index event were excluded. The primary outcome was a new diagnosis of SSc within 3 years of the index event. The 3-year observation window was selected based on available follow-up data in TriNetX; we note that this window may not capture late-onset cases, given that prior studies report a mean latency from LoS to SSc onset of 7.9 years (2, 3). Secondary outcomes included progressive systemic sclerosis, CREST syndrome, other/unspecified SSc and Raynaud syndrome. Six prespecified sensitivity analyses (S1–S6) were performed to assess robustness across different case definitions, time windows and patient subgroups (Appendix S1). Subgroup analyses for males, females and patients aged ≥50 and<50 were also performed.

Survival analysis was performed using Kaplan–Meier curves and Cox proportional hazards regression, with results expressed as hazard ratios (HR) with 95% confidence intervals (CI). Documented methotrexate treatment (RxNorm: 6851) served as a positive control outcome, as LoS patients are more likely to receive this treatment compared to controls (1, 4); replication of this known association confirms that the analytic pipeline can detect true effects (9). Burns and corrosions (ICD-10-CM: T20–T25) served as a negative control outcome to detect residual systematic bias (9). Analyses were performed using the TriNetX analytics platform.

## RESULTS

We included 58,826 LoS patients and an equal number of non-LoS controls (**Table I**). During follow-up, incident SSc was recorded in 1,478 LoS patients (2.5%) vs 22 controls (0.04%). LoS was strongly associated with subsequent SSc diagnosis, with hazard ratios exceeding 50 across different analyses (**Fig. 1**, Table SI). Results remained largely consistent across all sensitivity analyses. Importantly, exclusion of patients with positive antinuclear antibodies (S6) did not substantially attenuate the association (HR 58.0; 95% CI 36.4–92.4), providing strong evidence against the interpretation that findings are driven by misclassified SSc patients. Across SSc phenotypes, risks were similarly elevated with hazard ratios consistently exceeding 40–200. Raynaud syndrome also occurred more frequently after LoS, although with lower relative effect sizes, supporting the presence of early vasculopathic manifestations in a subset of patients (Table SI). The positive control (methotrexate) reproduced the expected strong association, supporting design validity (9). The negative control outcome showed null or near-null results across most sensitivity analyses; however, a statistically significant decrease in burns and corrosions was observed in the primary analysis and S1, indicating that residual systematic bias cannot be fully excluded in these analyses. The 2.5% absolute risk of SSc after LoS was consistent across all sensitivity analyses. In subgroup analyses, the association remained consistent with the primary analysis among females (HR 55.5) and patients aged ≥50 years (HR 44.7). Among patients aged <50 years, no SSc events occurred in the matched control group, precluding estimation of a hazard ratio; nonetheless, the risk difference was substantial (5.2%) with a highly significant log-rank test (*p*<0.001). The male subgroup yielded too few SSc events for a reliable estimate and could not be analysed (Table SII).

**Table I.** Baseline characteristics of study participants before and after propensity score matching

Characteristic	Definition (ICD-10-CM)	Before matching			After matching		
		Exposed *n*=64,521	Unexposed *n*=3,120,793	Std. diff.	Exposed *n*=58,826	Unexposed *n*=58,826	Std. diff.
Localized scleroderma							
Age at index (years, SD)	–	54.0±20.7	36.7±21.0	0.831	54.0±20.7	54.0±20.7	0.001
Female (%)	–	75.4	47.8	0.593	75.4	75.4	<0.001
American Indian or Alaska Native (%)	–	0.2	0.5	0.053	0.2	0.2	0.001
Asian (%)	–	1.7	4.0	0.139	1.7	1.7	0.002
Black or African American (%)	–	5.1	16.8	0.384	5.1	5.0	0.001
Hispanic or Latino (%)	–	5.1	8.3	0.128	5.1	5.0	0.001
White (%)	–	65.5	59.7	0.120	65.5	65.6	0.001
Persons with socioeconomic/psychosocial hazards (%)	Z55-Z65	2.0	4.3	0.131	2.0	2.0	0.002
Family history of musculoskeletal disease (%)	Z82.6	0.6	0.4	0.030	0.6	0.5	0.009
Occupational exposure (%)	Z57	0.1	0.1	0.015	0.1	0.1	0.005
Anxiety and related disorders (%)	F40-F48	16.7	16.6	0.004	16.7	16.7	<0.001
Nicotine dependence (%)	F17	4.6	9.6	0.197	4.6	4.6	0.001
Chronic kidney disease (%)	N18	3.6	3.5	0.007	3.6	3.6	0.002
Chronic lower respiratory diseases (%)	J40-J4A	14.2	14.1	0.002	14.2	14.2	0.001

ICD-10-CM:International Classification of Diseases, 10th Revision, Clinical Modification; LoS:localized scleroderma; SD:standard deviation; Std. diff.:standardized difference.

**Fig. 1.**
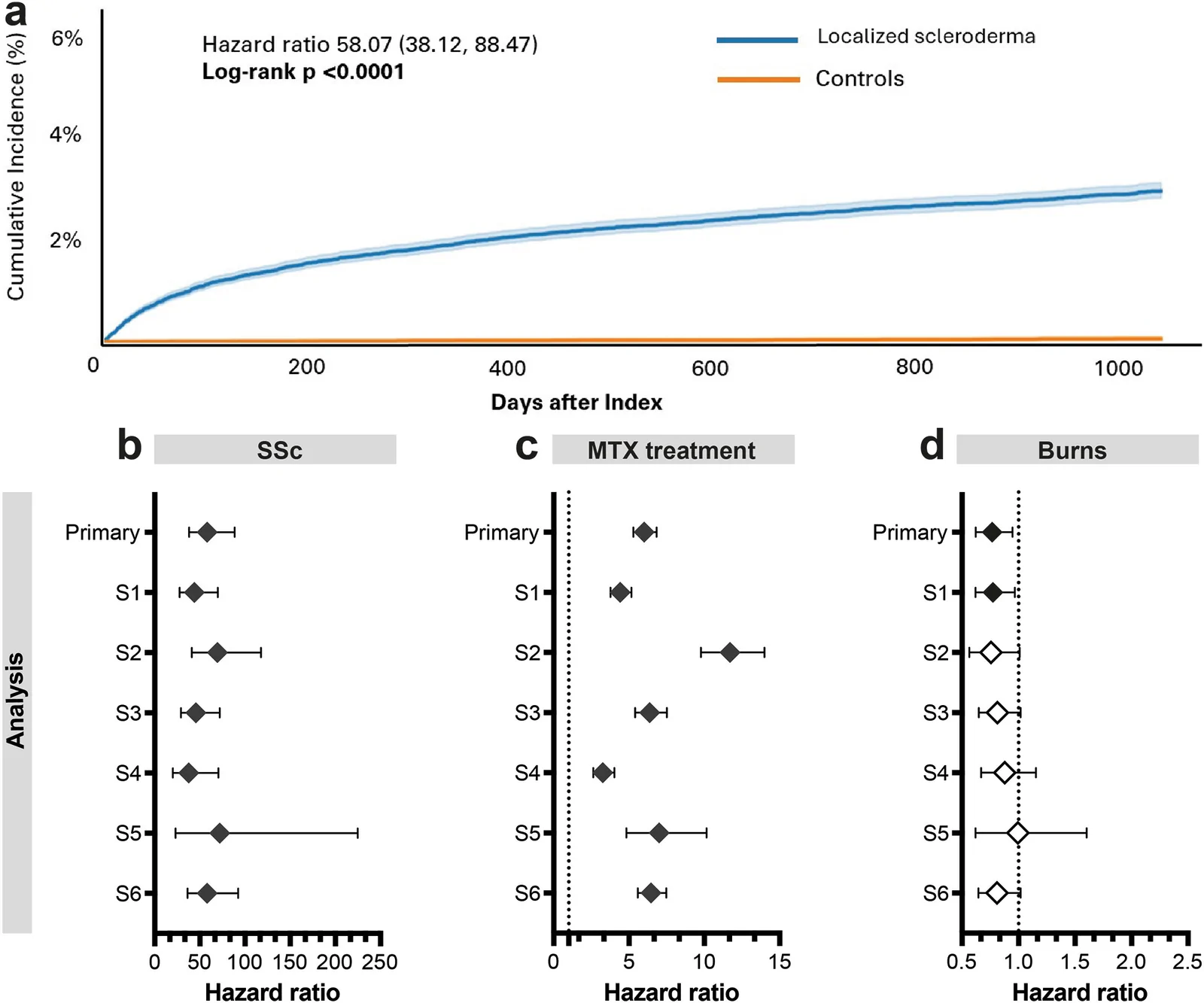
Localized scleroderma (LoS) is associated with an increased risk for systemic sclerosis (SSc). Outcomes were assessed from 1 day to 3 years after the index event in the primary analysis. Sensitivity analyses included: S1, outcomes from≥3 months after the index event; S2, second LoS documentation≥1 month later with matched control visit timing; S3,≥6 month minimum baseline window (MBW) to ensure baseline documentation; S4, outcomes from 1 to 3 years only; S5,≥3 LoS documentations; S6, excluding positive antinuclear antibodies (ANA) at any time before until 4 weeks after index. (a) Kaplan–Meier curves showing cumulative incidence of SSc in patients with LoS and matched controls over 3 years. (b–d) Forest plots display the hazard ratio (diamonds) and 95% confidence intervals (CIs) (error bars) for (b) SSc, (c) documented methotrexate (MTX) treatment as a positive control outcome, and (d) burns and corrosions as a negative control outcome. Dotted lines indicate a hazard ratio of one. Black diamonds indicate statistical significance; white diamonds indicate non-significant results.

## DISCUSSION

While the absolute 3 year risk of SSc following LoS was modest, the approximately 2.5% cumulative incidence represents a clinically meaningful excess compared with the very low background risk, particularly given the high disease burden of SSc. The magnitude of the HR should be interpreted in light of the near-absence of SSc in the control group (0.04%); when the background rate approaches zero, even a modest absolute risk yields a very large relative estimate. The clinically relevant measure is therefore the absolute excess risk of approximately 2.5% over 3 years, rather than the HR itself. The concern that findings could be driven by misclassification of SSc as LoS at baseline is substantially mitigated by the S6 sensitivity analysis, which excluded all patients with positive antinuclear antibodies and yielded a hazard ratio of 58.0 (95% CI 36.4–92.4), consistent with the primary analysis. These findings suggest that clinicians should consider systematic monitoring of LoS patients for early signs of systemic involvement, particularly during the first years after diagnosis.

Limitations include potential residual surveillance bias despite matching and possible misclassification of LoS and SSc. Because TriNetX is de-identified and federated, diagnoses cannot be attributed to a specific care setting or provider specialty and may derive from nonspecialist as well as specialist settings; however, requiring repeated LoS documentation (S2, S5) did not attenuate the association. The observation window was limited to approximately 3 years; given that prior studies report a mean latency of up to 7.9 years from LoS to SSc onset (2, 3), the current analysis likely underestimates the true long-term cumulative risk, and extended follow-up analyses are warranted. The statistically significant reduction in the negative control outcome observed in the primary analysis and S1 indicates that residual systematic bias in these analyses cannot be fully excluded. Unmeasured confounding, including genetic predisposition or broader autoimmune susceptibility, cannot be fully excluded.

## Data Availability

The data that support the findings of this study are available from the corresponding author upon reasonable request.

## Supplementary material

Figure S1.

Table SI.
