# Supplementary material for: Localized Scleroderma Is Associated with an Increased Risk of Systemic Sclerosis: A Matched Cohort Study
Source: Acta Derm Venereol. 2026 Oct 6;106:0759. doi: 10.2340/actadv.v106.adv-2026-0759 (PMC13639990; doi:10.2340/actadv.v106.adv-2026-0759)

Fig. S1. Flowchart of the study design.

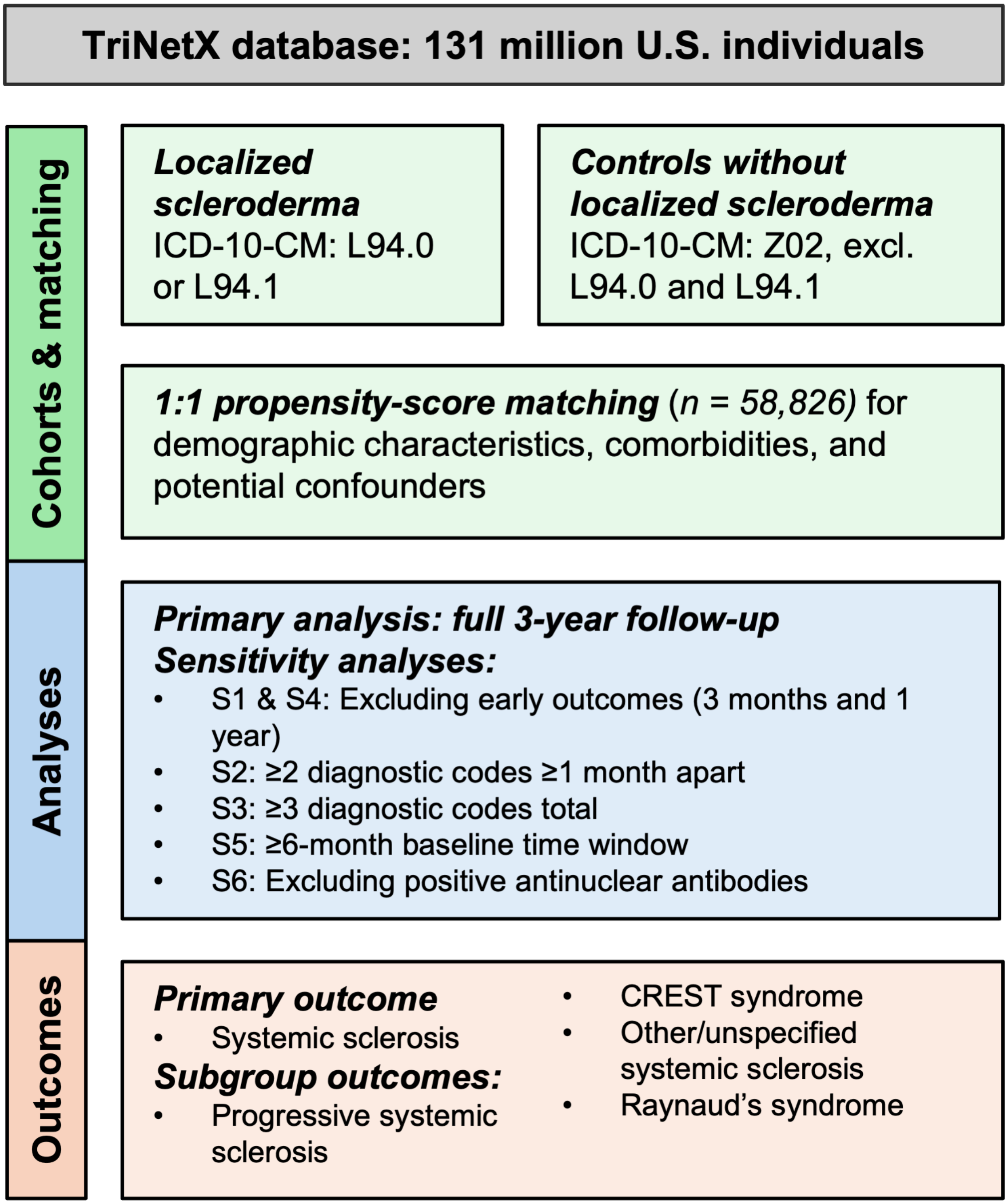

Supplement: Supplementary file 1 [file ActaDv-106-0759-s0001.pdf]
