# Supplementary material for: Localized Scleroderma Is Associated with an Increased Risk of Systemic Sclerosis: A Matched Cohort Study
Source: Acta Derm Venereol. 2026 Oct 6;106:0759. doi: 10.2340/actadv.v106.adv-2026-0759 (PMC13639990; doi:10.2340/actadv.v106.adv-2026-0759)
Supplement: Supplementary file 2 [file ActaDv-106-0759-s0002.pdf]

Table SI. Detailed results for all primary and secondary outcomes across the primary analysis and sensitivity analyses S1–S6. Outcomes were assessed from 1 day to 3 years after the index event in the primary analysis. Sensitivity analyses included: S1, outcomes from ≥3 months after the index event; S2, second LoS documentation ≥1 month later with matched control visit timing; S3, ≥6-month minimum baseline window; S4, outcomes from 1–3 years only; S5, ≥3 LoS documentations; S6, excluding positive antinuclear antibodies at any time before until 4 weeks after index.

HR: hazard ratio; CI: confidence interval; SSc: systemic sclerosis; MTX: methotrexate; LoS: localized scleroderma; CREST: calcinosis, Raynaud's phenomenon, esophageal dysmotility, sclerodactyly, telangiectasia.

Outcome	Exposed cohort (cases)			Unexposed cohort (comparators)			Hazard ratio (95% CI)	P-value
	N total	N outcomes	Risk (%)	N total	N outcomes	Risk (%)		
**Primary analysis (outcomes 1 day to 3 years)**								
Systemic sclerosis	58,826	1,478	2.51	58,826	22	0.04	58.072 (38.119–88.470)	<0.001
Progressive systemic sclerosis	58,826	643	1.09	58,826	10	0.02	184.630 (59.390–573.973)	<0.001
CREST syndrome	58,826	680	1.16	58,826	10	0.02	83.659 (39.732–176.154)	<0.001
Other/unspecified systemic sclerosis	58,826	1,448	2.46	58,826	17	0.03	73.599 (45.626–118.721)	<0.001
Raynaud’s syndrome	58,826	716	1.22	58,826	195	0.33	3.108 (2.653–3.642)	<0.001
Burns (negative control)	58,489	166	0.28	58,431	178	0.30	0.766 (0.620–0.947)	0.013
Methotrexate (positive control)	56,905	1,806	3.17	58,316	269	0.46	6.007 (5.285–6.828)	<0.001
**Sensitivity analysis S1 (outcomes 3 months to 3 years)**								
Systemic sclerosis	58,279	931	1.60	58,822	18	0.03	43.799 (27.473–69.828)	<0.001
Progressive systemic sclerosis	58,568	385	0.66	58,824	10	0.02	323.610 (45.469–2303.194)	<0.001
CREST syndrome	58,557	411	0.70	58,824	10	0.02	69.134 (28.622–166.983)	<0.001
Other/unspecified systemic sclerosis	58,290	912	1.56	58,823	14	0.02	55.126 (32.518–93.453)	<0.001
Raynaud’s syndrome	58,637	527	0.90	58,769	138	0.23	3.164 (2.624–3.817)	<0.001
Burns (negative control)	58,473	150	0.26	58,411	158	0.27	0.773 (0.619–0.967)	0.024
Methotrexate (positive control)	56,040	941	1.68	58,232	185	0.32	4.411 (3.767–5.164)	<0.001
**Sensitivity analysis S2 (≥2 diagnoses ≥1 month apart)**								

Systemic sclerosis	28,095	1,019	3.63	28,095	14	0.05	69.491 (41.009–117.756)	<0.001
Progressive systemic sclerosis	28,095	467	1.66	28,095	10	0.04	88.974 (36.860–214.769)	<0.001
CREST syndrome	28,095	482	1.72	28,095	10	0.04	91.858 (38.060–221.697)	<0.001
Other/unspecified systemic sclerosis	28,095	1,004	3.57	28,095	13	0.05	73.715 (42.653–127.399)	<0.001
Raynaud's syndrome	28,095	379	1.35	28,095	86	0.31	4.078 (3.227–5.154)	<0.001
Burns (negative control)	27,940	84	0.30	27,882	100	0.36	0.756 (0.565–1.010)	0.058
Methotrexate (positive control)	26,821	1,500	5.59	27,786	130	0.47	11.697 (9.778–13.993)	<0.001
**Sensitivity analysis S3 (minimum baseline time window of ≥6 months)**								
Systemic sclerosis	44,796	943	2.10	44,796	19	0.04	45.690 (29.013–71.956)	<0.001
Progressive systemic sclerosis	44,796	351	0.78	44,796	10	0.02	321.002 (45.091–2285.190)	<0.001
CREST syndrome	44,796	383	0.86	44,796	10	0.02	70.005 (28.972–169.153)	<0.001
Other/unspecified systemic sclerosis	44,796	922	2.06	44,796	15	0.03	56.561 (33.959–94.207)	<0.001
Raynaud's syndrome	44,796	535	1.19	44,796	147	0.33	3.295 (2.745–3.955)	<0.001
Burns (negative control)	44,459	145	0.33	44,396	158	0.36	0.813 (0.649–1.019)	0.071
Methotrexate (positive control)	43,505	1,092	2.51	44,211	161	0.36	6.370 (5.399–7.517)	<0.001
**Sensitivity analysis S4 (outcomes 1-3 years)**								
Systemic sclerosis	59,210	459	0.78	60,222	10	0.02	37.636 (20.114–70.420)	<0.001
Progressive systemic sclerosis	59,768	181	0.30	60,236	10	0.02	73.113 (18.146–294.583)	<0.001
CREST syndrome	59,744	194	0.32	60,232	10	0.02	52.309 (16.724–163.608)	<0.001
Other/unspecified systemic sclerosis	59,232	451	0.76	60,225	10	0.02	41.046 (21.218–79.401)	<0.001
Raynaud's syndrome	59,821	305	0.51	60,118	79	0.13	3.116 (2.433–3.991)	<0.001
Burns (negative control)	59,830	108	0.18	59,737	98	0.16	0.878 (0.668–1.154)	0.349
Methotrexate (positive control)	56,850	413	0.73	59,551	106	0.18	3.255 (2.629–4.029)	<0.001
**Sensitivity analysis S5 (≥3 diagnoses in total)**								
Systemic sclerosis	20,004	292	1.46	20,584	10	0.05	72.063 (23.107–224.737)	<0.001
Progressive systemic sclerosis	20,295	114	0.56	20,588	10	0.05	41.346 (10.216–167.334)	<0.001
CREST syndrome	20,282	118	0.58	20,585	10	0.05	42.871 (10.597–173.436)	<0.001
Other/unspecified systemic sclerosis	20,015	290	1.45	20,587	10	0.05	71.555 (22.943–223.164)	<0.001
Raynaud's syndrome	20,423	134	0.66	20,550	27	0.13	3.552 (2.349–5.371)	<0.001
Burns (negative control)	20,454	41	0.20	20,434	29	0.14	0.995 (0.619–1.602)	0.985

Methotrexate (positive control)	18,344	277	1.51	20,334	31	0.15	7.000 (4.829–10.146)	<0.001
**Sensitivity analysis S6 (excluding positive antinuclear antibodies)**								
Systemic sclerosis	49,360	1,188	2.41	49,360	18	0.04	58.002 (36.416–92.382)	<0.001
Progressive systemic sclerosis	49,360	474	0.96	49,360	10	0.02	207.259 (51.684–831.143)	<0.001
CREST syndrome	49,360	504	1.02	49,360	10	0.02	110.166 (41.187–294.671)	<0.001
Other/unspecified systemic sclerosis	49,360	1,162	2.35	49,360	17	0.03	60.055 (37.205–96.940)	<0.001
Raynaud's syndrome	49,360	569	1.15	49,360	174	0.35	2.820 (2.380–3.343)	<0.001
Burns (negative control)	49,063	145	0.30	49,039	151	0.31	0.810 (0.645–1.018)	0.070
Methotrexate (positive control)	47,899	1,479	3.09	48,934	208	0.43	6.460 (5.587–7.469)	<0.001

Table SII. Results for subgroup analyses for the primary outcome of systemic sclerosis.

CI: confidence interval

*Male stratum: too few systemic sclerosis events for a stable estimate; analysis not performed. Counts not shown.

[†]No systemic sclerosis events among matched controls, so hazard ratio/CI (and risk ratio/odds ratio) are not estimable.

**Subgroup**	**Exposed cohort (cases)**			**Unexposed cohort (comparators)**			**Hazard ratio (95% CI)**	**P-value**
	**N total**	**N outcomes**	**Risk (%)**	**N total**	**N outcomes**	**Risk (%)**		
**Sex**								
Female	40,873	1,287	3.15	40,873	21	0.05	55.544 (36.089–85.486)	<0.001
Male*	-	-	-	-	-	-	Not estimable	-
**Age**								
≥50 years	34,986	975	2.79	34,986	20	0.06	44.744 (28.738–69.665)	<0.001
<50 years	12,965	676	5.21	12,965	0	0	Not estimable[†]	<0.001[†]
