# Supplementary material for: Localized Scleroderma Is Associated with an Increased Risk of Systemic Sclerosis: A Matched Cohort Study
Source: Acta Derm Venereol. 2026 Oct 6;106:0759. doi: 10.2340/actadv.v106.adv-2026-0759 (PMC13639990; doi:10.2340/actadv.v106.adv-2026-0759)
Supplement: Supplementary file 3 [file ActaDv-106-0759-s0003.pdf]

Appendix S1

Supplementary methods

Ethics

The data reviewed is a secondary analysis of existing data, does not involve intervention or interaction with human subjects, and is de-identified per the de-identification standard defined in Section §164.514(a) of the HIPAA Privacy Rule. TriNetX is a federated network which facilitates collaboration between industry including pharmaceutical companies and research organizations, academia, health care organizations (HCOs). TriNetX has a security and governance model that provides a federated platform of real-world-data (RWD, i.e., electronic health records (EHRs) which is Health Insurance Portability and Accountability Act (HIPAA), General Data Protection Regulation (GDPR), and Lei Geral de Proteção de Dados (LGPD)-compliant. Thus, our study did not require Institutional Review Board approval. In addition, the use of the TriNetX platform for research was approved by the Swedish Ethical Review Authority (dnr 2025-03805-02).

Study design and database

A propensity-score matched (PSM) retrospective cohort study was conducted using the US Collaborative Network from the federated EHR database TriNetX, following established protocols (1–4). This network was selected due to the large number of documented EHRs and the high degree of covariate documentation (5). Through a cooperation with TriNetX, UKSH researchers have access to the TriNetX database and analytical platform. We retrieved EHRs with a diagnosis of localized scleroderma (LoS) and non-LoS controls. Systemic sclerosis (SSc) was the primary outcome. SSc-subtypes (progressive systemic sclerosis, CREST syndrome, other/unspecified SSc), and Raynaud's syndrome) were secondary endpoints. Documentation of methotrexate (MTX), used for LoS treatment (6) served as positive control outcome, whilst ICD-10-CM:T20-T25 (burns and corrosions of external body surface) served as a negative control outcome. The analytical framework, definition of cohorts, matching factors, and outcomes were defined prior to data collection (**Supplementary Figure 1**). The study was performed according to the STROBE guidelines (7).

Study population

The study was conducted in August 2025. At the time of analysis, access to over 150 million patients across healthcare organizations worldwide was provided. LoS was defined by documentation of ICD-10-CM:L94.0 or L94.1. Non-LoS controls were retrieved based on the absence of any of these codes and a documented healthcare

visit for administrative examination (ICD-10-CM:Z02). We selected Z02 to ensure recent healthcare contact and sufficient baseline data capture for propensity score matching. We acknowledge that this approach may introduce healthcare utilization bias. Alternative encounter codes (e.g., Z00) were considered; however, the resulting cohort size exceeded computational limits for robust propensity score matching. Thus, cohort definition reflected a methodological trade-off between minimizing utilization bias and maintaining feasible, high-quality matching.For both groups, EHRs with a diagnostic for Raynaud's syndrome (ICD-10-CM:I73.0) or SSC (ICD-10-CM:M34) prior to the index event were excluded. Index events were defined by the date of LoS diagnosis or, for controls, the date of the healthcare encounter.

Covariates

To minimize bias from potentially confounding variables, PSM was performed by establishing a covariate matrix that included demographic information, potential health hazards related to socioeconomic and psychosocial circumstances, and relevant concomitant diagnoses. Covariates included in all analyses were age at index (continuous), female sex (binary), self-reported ancestry (binary), and putative risk factors of SSc (**Table I**). Matching was performed 1:1 using propensity-scores. Baseline characteristics were reported before and after matching (**Table I**).

Outcomes

Primary and secondary outcomes were defined by the following ICD-10-CM codes: M34 (any SSc), M34.0 (progressive systemic sclerosis), M34.1 (CREST syndrome), M34.8 or M34.9 (other and unspecified forms of systemic sclerosis), and I73.0 (Raynaud's syndrome). Negative and positive control outcomes were included to assess potential bias and validate the analytic approach. Burns and corrosions (ICD-10-CM:T20–T25) were selected as a negative control outcome, as no association with LoS was expected. A null finding would argue against general coding or measurement bias. Methotrexate (RxNorm: 6851) was selected as a positive control outcome, as LoS patients are more likely to receive this treatment compared to controls. Replication of this known association would confirm that the study design and analytic pipeline can detect true effects. Both controls were analyzed using the same PSM cohorts and statistical models applied to the other outcomes. Any outcomes, except the negative and positive controls, occurring prior to the index event were excluded during data retrieval. For negative and positive control outcomes, the default option of the TriNetX analytical platform was used that excludes EHRs with the outcome after PSM.

Primary and sensitivity analyses

In the primary analysis, outcomes occurring any time from one day to three years after the index event were considered. In sensitivity analysis S1, to exclude already present but nor diagnosed or coded outcomes at the index event, as well as to counter reverse causality, outcomes occurring between three months to three years after the index event were considered. In S2, a second documentation of LoS at least one month after its first documentation was required. To achieve a similar temporal structure between groups, controls were required to have a documented visit to the HCO at least one month after the initial healthcare encounter for administrative examination. For S3, a minimal baseline time window (MBW) of six months was mandated to ensure documentation of baseline characteristics and further mitigate detection bias. S4, to further minimize the risk of misclassification, only considered outcomes occurring from 1 year to 3 years after the index event. S5 mandated at least three or more documentations of LoS. In S6 all EHRs with documented positive antinuclear antibodies at any time before until 4 weeks after the index event were excluded from both groups. Across all analysis, the index event was defined by the initial documentation of LoS or healthcare encounter.

Statistical analysis

A propensity-score for each patient was generated by logistic regression analysis (with exposure as the dependent variable) using the Python package scikit-learn. Matching was performed 1:1 using the greedy nearest neighbor approach with a cut-off distance of 0.1 pooled standard deviations of the logit of the propensity-score. Baseline characteristics were re-evaluated and reported after matching, differences were compared by t-test for continuous and z-test for binary or categorical variables. Relative risks and risk difference (RD) were calculated. Survival analysis was performed using the Kaplan-Meier method (KM) in Survival package v3.2-3 in R (R Foundation for Statistical Computing, Vienna, Austria) and validated by comparison with the outputs of SAS version 9.4 (SAS, Cary, NC). The proportionality assumption was tested by the coxph function in R's Survival package. KM-curves were compared using the Log-rank test. A univariate Cox proportional hazards regression was used to express hazard ratios (HR)s with 95%-confidence intervals (CI)s.

Use of artificial intelligence

ChatGPT (OpenAI, San Francisco, CA; GPT-5) was used for language editing of the manuscript. The authors reviewed all suggested changes, made further edits, and take full responsibility for the content.

Data sharing

All data relating to the retrospective cohort studies are included in the manuscript or the supplementary tables.

Supplementary references

1. Ludwig RJ, Anson M, Zirpel H, Thaci D, Olbrich H, Bieber K, et al. A comprehensive review of methodologies and application to use the real-world data and analytics platform TriNetX. Front Pharmacol. 2025 Feb 13;16:1516126.

2. Kolkhir P, Bieber K, Hawro T, Kridin K, Ludwig MA, Olbrich H, et al. Mortality in adult patients with chronic spontaneous urticaria: A real-world cohort study. J Allergy Clin Immunol. 2024 Dec 13;S0091-6749(24)02355-8.

3. Kridin K, Bieber K, Vorobyev A, Moderegger EL, Olbrich H, Ludwig MA, et al. Biological, as opposed to classic antipsoriatic drug or apremilast, treatment mitigates the risk of death and cardiovascular disease in psoriasis. EBioMedicine. 2025 Jan;111:105485.

4. Curman P, Ngo BT, Ludwig RJ, Kasperkiewicz M. Bidirectional risk relationship between autoimmune blistering diseases and monoclonal gammopathies: A large-scale matched cohort study. JAAD Int. 2025 Oct;22:37–9.

5. Palchuk MB, London JW, Perez-Rey D, Drebert ZJ, Winer-Jones JP, Thompson CN, et al. A global federated real-world data and analytics platform for research. JAMIA Open. 2023 July;6(2):ooad035.

6. Papara C, De Luca DA, Bieber K, Vorobyev A, Ludwig RJ. Morphea: The 2023 update. Front Med Lausanne. 2023;10:1108623.

7. von Elm E, Altman DG, Egger M, Pocock SJ, Gøtzsche PC, Vandenbroucke JP, et al. The Strengthening the Reporting of Observational Studies in Epidemiology (STROBE) statement: guidelines for reporting observational studies. Lancet Lond Engl. 2007 Oct 20;370(9596):1453–7.
